# *Plasmodium vivax* Cell-Traversal Protein for Ookinetes and Sporozoites: Naturally Acquired Humoral Immune Response and B-Cell Epitope Mapping in Brazilian Amazon Inhabitants

**DOI:** 10.3389/fimmu.2017.00077

**Published:** 2017-02-07

**Authors:** Rodrigo Nunes Rodrigues-da-Silva, Isabela Ferreira Soares, Cesar Lopez-Camacho, João Hermínio Martins da Silva, Daiana de Souza Perce-da-Silva, Antônio Têva, Antônia Maria Ramos Franco, Francimeire Gomes Pinheiro, Lana Bitencourt Chaves, Lilian Rose Pratt-Riccio, Arturo Reyes-Sandoval, Dalma Maria Banic, Josué da Costa Lima-Junior

**Affiliations:** ^1^Laboratory of Immunoparasitology, Oswaldo Cruz Institute, Fiocruz, Rio de Janeiro, Brazil; ^2^Nuffield Department of Medicine, The Jenner Institute, The Henry Wellcome Building for Molecular Physiology, University of Oxford, Oxford, UK; ^3^Computational Modeling Group, Fiocruz, Fortaleza, Brazil; ^4^Laboratory of Clinical Immunology, Oswaldo Cruz Institute, Fiocruz, Rio de Janeiro, Brazil; ^5^Laboratory of Immunodiagnostics, Department of Biological Sciences, National School of Public Health, Fiocruz, Rio de Janeiro, Brazil; ^6^Laboratory of Leishmaniasis and Chagas Disease, National Institute of Amazonian Research, Manaus, Brazil; ^7^Laboratory of Malaria Research, Oswaldo Cruz Institute, Fiocruz, Rio de Janeiro, Brazil

**Keywords:** PvCelTOS, *P. vivax*, vaccines, epitope mapping, epitope prediction, malaria vaccines, malaria

## Abstract

The cell-traversal protein for ookinetes and sporozoites (CelTOS), a highly conserved antigen involved in sporozoite motility, plays an important role in the traversal of host cells during the preerythrocytic stage of *Plasmodium* species. Recently, it has been considered an alternative target when designing novel antimalarial vaccines against *Plasmodium falciparum*. However, the potential of *Plasmodium vivax* CelTOS as a vaccine target is yet to be explored. This study evaluated the naturally acquired immune response against a recombinant *P. vivax* CelTOS (PvCelTOS) (IgG and IgG subclass) in 528 individuals from Brazilian Amazon, as well as the screening of B-cell epitopes *in silico* and peptide assays to associate the breadth of antibody responses of those individuals with exposition and/or protection correlates. We show that PvCelTOS is naturally immunogenic in Amazon inhabitants with 94 individuals (17.8%) showing specific IgG antibodies against the recombinant protein. Among responders, the IgG reactivity indexes (RIs) presented a direct correlation with the number of previous malaria episodes (*p* = 0.003; *r* = 0.315) and inverse correlation with the time elapsed from the last malaria episode (*p* = 0.031; *r* = −0.258). Interestingly, high responders to PvCelTOS (RI > 2) presented higher number of previous malaria episodes, frequency of recent malaria episodes, and ratio of cytophilic/non-cytophilic antibodies than low responders (RI < 2) and non-responders (RI < 1). Moreover, a high prevalence of the cytophilic antibody IgG1 over all other IgG subclasses (*p* < 0.0001) was observed. B-cell epitope mapping revealed five immunogenic regions in PvCelTOS, but no associations between the specific IgG response to peptides and exposure/protection parameters were found. However, the epitope (PvCelTOS_I136-E143_) was validated as a main linear B-cell epitope, as 92% of IgG responders to PvCelTOS were also responders to this peptide sequence. This study describes for the first time the natural immunogenicity of PvCelTOS in Amazon individuals and identifies immunogenic regions in a full-length protein. The IgG magnitude was mainly composed of cytophilic antibodies (IgG1) and associated with recent malaria episodes. The data presented in this paper add further evidence to consider PvCelTOS as a vaccine candidate.

## Introduction

Malaria remains a major public health problem worldwide. It is caused by protozoan parasites of the genus *Plasmodium*, being responsible for nearly 438,000 deaths and 150–300 million new infections in 2015 (1) and the reason of enormous socioeconomic impact in endemic settings (2). Among the *Plasmodium* species able to infect humans, *Plasmodium falciparum* and *Plasmodium vivax* are the most prevalent malaria parasites. *P. falciparum* is extremely prevalent in Africa and is responsible for the majority of cases and deaths worldwide, while *P. vivax* is the most prevalent species outside Africa (3). Despite the reduction in the number of malaria cases and deaths over the past decade (1), the emergence of drug resistance and the significant ongoing burden of morbidity and mortality emphasize the need for an effective malaria vaccine. Unfortunately, potential *P. vivax* vaccine candidates lag far behind those for *P. falciparum* (4). Currently, besides the RTS, S vaccine, there are 30 candidate vaccine formulations in clinical trials against *P. falciparum*, while there is only one against *P. vivax* (5). These data allied to the impact caused by the high *P. vivax* prevalence (2), the severity of the disease (6–11), and the emergence of strains resistant to chloroquine (12–14) and primaquine (15–17), reiterate the importance of identifying and exploring the potential of vaccine candidates against *P. vivax* as an essential step in the development of a safe and affordable vaccine.

Malaria liver-stage vaccines are one of the leading strategies and the only approach that has demonstrated complete, sterile protection in clinical trials. Therefore, vaccines targeting sporozoite and liver-stage parasites, when parasite numbers are low, can lead to the elimination of the parasite before it advances to the symptomatic stage of the disease (18). Corroborating this idea, the sterile protection against *P. falciparum* by immunization with radiation-attenuated sporozoites was demonstrated in several studies (19–21) and the protection lasted for at least 10 months and extended to heterologous strain parasites (22). Based on these findings, sporozoite surface antigens are one of the most promising vaccine targets against malaria, to protect and prevent the symptoms and block its transmission. To date, RTS,S, the subunit vaccine consisting of a portion of *P. falciparum* circumsporozoite protein (CSP), conferred partial protection in Phase III trials and fell short of community-established vaccine efficacy goals (23–26). Conversely, Gruner and collaborators have demonstrated that the sterile protection against sporozoites can be obtained in the absence of specific immune responses to CSP (27). In addition, a recent study found 77 parasite proteins associated with sterile protection against irradiated sporozoites (28). Collectively, these data reinforce the concept that a multivalent anti-sporozoite vaccine targeting several surface-exposed antigens would induce a higher protection efficacy.

In this scenario, cell-traversal protein of *Plasmodium* ookinetes and sporozoites, a highly conserved protein among *Plasmodium* species, emerged as a novel target in the development of a vaccine against *Plasmodium* parasites (29). This secretory microneme protein is translocated to the sporozoites and ookinetes surface, being necessary for sporozoites and ookinetes to break through cellular barriers and establish infection in the new host, having a crucial role on cell-transversal ability in both stages (29, 30). The disruption of the genes encoding CelTOS in *Plasmodium berghei* reduces the infectivity in the mosquito host and also the infectivity of the sporozoite in the liver, almost eliminating their ability to cell pass (29). Interestingly, *P. falciparum* CelTOS (PfCelTOS) was naturally recognized by acquired antibodies in exposed populations (31), able to induce cross-reactive immunity against *P. berghei* and inhibit sporozoite motility and invasion of hepatocytes *in vitro* (32). However, the knowledge about *P. vivax* CelTOS (PvCelTOS) has remained limited. Only recently, a study reported PvCelTOS as naturally immunogenic in infected individuals from Western Thailand. Our group, investigating the genetic diversity of genes encoding PvCelTOS in field isolates from five different regions of the Amazon forest, reveals a high-conserved profile. Together, both findings support the potential of PvCelTOS as an interesting target on *P. vivax* sporozoite surface, but further studies are still necessary to consolidate this protein as an alternative in future multitarget vaccines. Therefore, the present study aimed at evaluating the naturally acquired humoral immune response against PvCelTOS in exposed populations from Brazilian Amazon, determining the antibody subclass profile, identifying its B-cell epitopes and verifying the existence of associations between the specific IgG and subclass response against PvCelTOS and epidemiological data that can reflect the exposition and/or protection degree.

## Participants and Methods

### Study Area and Volunteers

A cross-sectional cohort study was conducted involving 528 individuals from Rio Preto da Eva (2°50′50″S/59°56′28″W), located north of the Amazon River and 80 km distant from Manaus, the capital of Amazon state. This city has an area of 6,000 km^2^ and a population of about 22,000 people, who live in rural areas inside the forests. Transmission of malaria in the Amazon occurs throughout the whole year, with seasonal fluctuations with maximum transmission occurring during the dry season from May to October and prevalence of infections by *P. vivax*, responsible for more than 85% of reported malaria cases.

Samples and survey data were collected from November 2013 to March 2015. In addition, we also included, as control subjects, 10 naive individuals living in Manaus, and with no reported previous malaria episodes. Written informed consent was obtained from all adult donors or from parents of donors in the case of children. The study was reviewed and approved by the Fundação Oswaldo Cruz Ethical Committee and the National Ethical Committee of Brazil.

### Epidemiological Survey

In order to evaluate the possible influence of epidemiological factors on humoral immunity against PvCelTOS, all donors were interviewed upon informed consent prior to blood collection. The survey included questions related to personal exposure to malaria, such as years of residence in the endemic area, recorded individual and family previous malaria episodes, use of malaria prophylaxis, presence/absence of symptoms, and personal knowledge of malaria transmission. All epidemiological data were stored in Epi-Info for subsequent analysis (Centers for Disease Control and Prevention, Atlanta, GA, USA).

### Malaria Diagnosis and Blood Sampling

Venous peripheral blood was drawn into heparinized tubes and plasma collected after centrifugation (350 × *g*, 10 min). Plasma samples were stored at −20°C and transported to our laboratory. Thin and thick blood smears of all donors were examined for malaria parasites. Parasitological evaluations were done by examination of 200 fields at 1,000× magnification under oil-immersion and a research expert in malaria diagnosis examined all slides. Donors positive for *P. vivax* and/or *P. falciparum* at the time of blood collection were subsequently treated using the chemotherapeutic regimen recommended by the Brazilian Ministry of Health.

### Recombinant PvCelTOS Expression in HEK-293T Cells

As previously described (33), the *P. vivax* sequence for CelTOS (Salvador I; Uniprot accession number Q53UB7) was cloned in the expression vector pHLsec, which is flanked by the chicken β-actin/rabbit β-globin hybrid promoter with a signal secretion sequence and a Lys-His6 tag. The protein was expressed upon transient transfection in HEK-293T cells with endotoxin-free plasmids in roller bottles (2,125 cm^2^). The secreted protein was purified from the supernatant by immobilized Ni Sepharose affinity chromatography. The presence of proteins in the elution samples was confirmed using 6xHis epitope tag antibody [horseradish peroxidase (HRP) conjugate] in a Western blot. The sample was concentrated using an Amicon Ultra centrifugal filter system (Life Technologies) until reaching 10 ml of final volume. Contaminant proteins and salts were removed from the concentrate by size exclusion purification (SEC) using Superdex medium in the column. Protein concentration after recovery was tested using a Bradford protein assay, and purity was assessed by silver staining and by Western blotting.

### Antibody Assays

Anti-PvCelTOS specific antibodies were evaluated on plasma samples from 528 exposed individuals from Brazilian Amazon and 10 healthy individuals, who had no reported malaria episodes, using enzyme-linked immunosorbent assay (ELISA), essentially as previously described (33, 34). Briefly, MaxiSorp 96-well plates (Nunc, Rochester, NY, USA) were coated with PBS containing 1.5 µg/ml of recombinant protein. After overnight incubation at 4°C, the plates were washed and blocked for 1 h at 37°C. Individual plasma samples diluted 1:100 in PBS-Tween containing 5% non-fat dry milk (PBS-Tween-M) were added in duplicate wells. After 1 h at 37°C and three washings with PBS-Tween, bound antibodies were detected with peroxidase-conjugated goat antihuman IgG (Sigma, St. Louis) and followed by addition of *o*-phenylenediamine and hydrogen peroxide. Optical density was identified at 492 nm using a SpectraMax 250 ELISA reader (Molecular Devices, Sunnyvale, CA, USA). The results for total IgG were expressed as reactivity indexes (RIs), which were calculated by the mean optical density of an individual’s tested sample divided by the mean optical density of 10 non-exposed control individuals’ samples plus 3 standard deviations. Subjects were scored as responders to PvCelTOS if the RI of IgG against the recombinant protein was higher than 1. Additionally, the RIs of IgG subclasses were evaluated on responders individuals by a similar method, using peroxidase-conjugated goat antihuman IgG1, IgG2, IgG3, and IgG4 (Sigma, St. Louis).

### B Cell Epitope Prediction on PvCelTOS

The prediction of linear B-cell epitopes was carried out using the program BepiPred (35), which is based on hidden Markov model profiles of known antigens, and also incorporates hydrophilicity and secondary structure prediction. For each input FASTA sequence, the server outputs a prediction score for each amino acid. The recommended cutoff of 0.35 was used to determine potential B-cell linear epitopes, ensuring sensibility of 49% and specificity of 75% to this approach. Linear B-cell epitopes are predicted to be located at the residues with the highest scores. In this study, BepiPred was used to predict B-cell linear epitopes and to evaluate the prediction value of peptides containing short amino acid sequences of PvCelTOS.

The Emini surface accessibility (ESA) was used to evaluate the probability of predicted linear B-cell epitopes to be exposed on the surface of the protein. This approach calculates the surface accessibility of hexapeptides and values greater than 1.0 indicate an increased probability of being found on the surface (36). Sequences with BepiPred score above 0.35 and ESA score above 1.0 were considered potential linear B-cell epitopes in regions that could be accessed by naturally acquired antibodies.

### B-Cell Epitope Mapping of PvCelTOS

A peptide library of 32 PvCelTOS synthetic 15-mer peptides overlapping by nine amino acids (GenOne Biotechnologies; purity 95% based on HPLC) was synthesized. To evaluate the specific response to each peptide, the peptide array was performed using MaxiSorp 96-well plates (Nunc, Rochester, NY, USA) coated with PBS containing 5 µg/ml of each peptide in duplicates. After overnight incubation at 4°C, the plates were washed with PBS and blocked with PBS-Tween containing 5% non-fat dry milk (PBS-Tween-M) for 1 h at 37°C. Individual plasma samples were diluted 1:100 with PBS-Tween-M and added in duplicate wells for each sequence and the plates incubated at room temperature for 1 h. After three washings with PBS-Tween, bound antibodies were detected with peroxidase-conjugated goat antihuman IgG (Sigma, St. Louis) followed by addition of *o*-phenylenediamine and hydrogen peroxide. The absorbance was read at 492 nm using an ELISA reader (Spectramax 250, Molecular Devices, Sunnyvale, CA, USA). The results for total IgG were expressed as RIs, which were calculated by the mean optical density of the tested samples plus 3 standard deviations of pools of control individuals. Subjects were scored as positive if the RI was higher than 1.

### Root Mean Square Fluctuation (RMSF) and Electrostatic Potential Surface Calculation

Molecular dynamics simulations were carried out using GROMACS 5.1.2 (37) software package. Gromos53a6 (38) force field was used. Simple point charge water model (39) was used to solvate the system. Charges were neutralized using Na+ and Cl− ions. Steepest descent method was used for energy minimization. Further, 100 ps temperature equilibration was carried out at a temperature of 300 K in the presence of position restraints of 1,000 KJ/mol and the pressure coupling of 1,000 ps at 1 bar of atmospheric pressure. After equilibration, the simulation of 200,000 ps (200 ns) without position restraints was carried out. All simulations were run three times, and consistent results were recorded. RMSF was analyzed from simulation trajectory using GROMACS utilities. The Electrostatic potential surface for the PvCelTOS was calculated using APBS (40) and visualized in PyMOL (Pymol LLC) and the electrostatic potential surfaces for the contours from −3kT/e (red) to +3kT/e (blue) were visualized. The figures were rendered using PyMol.

### Statistical Analysis

All statistical analyzes were carried out using Prism 5.0 for Windows (GraphPad Software, Inc.). The one-sample Kolmogorov–Smirnoff test was used to determine whether a variable was normally distributed. The Mann–Whitney test was used to compare RIs of IgG against recombinant PvCelTOS between studied groups. Differences in proportions of the RI of IgG subclasses and epidemiological parameters were evaluated by Fisher’s exact test and associations between antibody responses and epidemiological data were determined by Spearman rank test. A two-sided *p* value <0.05 was considered significant.

## Results

### Epidemiological Profile of Studied Individuals

Most studied individuals were adults and naturally exposed to malaria infection throughout the years (Table 1). Age ranged from 10 to 89 years with an average of 36.9. The proportion of men was significantly higher (53.8%) than for women (46.2%; χ^2^ = 5.761, *p* < 0.0164). Regarding the previous personal history of malaria, only seven individuals reported no malaria episode (1.3%). Among those who remembered the *Plasmodium* species, the majority (29.9%) reported infections by *P. falciparum* and *P. vivax*. The number of past malaria episodes also varied greatly among donors, ranging from 0 to 50 (mean = 7.74 ± 16.5). Finally, the time elapsed since the last malaria episode ranged from 0 to 480 months (mean = 71.7 ± 77.9). Interestingly, a correlation trend was observed between the time of residence in the endemic area and the number of previous malaria infections (*p* = 0.0003; *r* = 0.153). Collectively, the epidemiological inquiry indicated that the studied population had different degrees of exposure and/or immunity.

**Table 1:** **Summary of the epidemiological data of the studied population**.

	Overall	PvCelTOS IgG responders	PvCelTOS IgG non-responders (NRs)
**Gender—***N*** (%)**			
Male	284 (53.8%)	55 (58.5%)	229 (52.8%)
Female	244 (46.2%)	39 (41.5%)	205 (47.2%)
Total	528	94	434
**Malaria exposure—median (IR)**			
Age (years)	36 (25–50)	38 (21–55.5)	36 (21–50)
Time of residence in endemic area (years)	33 (19–49)	35 (21–55)	33 (19–48)
Number of previous malaria episodes (*n*)	4 (2–10)	4.5 (2–10)	4 (2–10)
Time since the last malaria episode (months)	51 (24–91)	60 (13.7–89.2)	51 (24–90.5)
Frequency of recent malaria episodes (%)	12.7%	16.0%	13.1%
**Previous malaria species contracted—***N*** (%)**			
Never infected	7 (1.3%)	0 (0%)	7 (1.6%)
*Plasmodium falciparum*	32 (6.1%)	5 (5.3%)	27 (6.2%)
*Plasmodium vivax*	125 (23.7%)	25 (26.6%)	100 (23%)
Both species	158 (29.9%)	31 (33%)	127 (29.3)
Not reported/remember	206 (39%)	33 (35.1%)	173 (39.9%)

*Values of age, time of residence in endemic areas, number of previous malaria episodes, and time elapsed from the last malaria episode represent the median (interquartile range), while the parameter “frequency of recent malaria episodes” represents the percentage of individuals who reported malaria episode in the last year. The frequency of individuals who present recent malaria episodes was compared by Fisher’s test, and other epidemiological parameters were compared by Mann–Whitney test. No statistical difference was observed between epidemiological parameters of responders and NR individuals*.

### PvCelTOS Is Naturally Immunogenic with the Prevalence of Cytophilic Antibodies in Brazilian Amazon Individuals

To test if the PvCelTOS is a target for naturally acquired antibodies in Amazon individuals, we first assessed the IgG reactivity profile against the recombinant protein. The plasma samples collected from the 528 individuals living in the endemic area reveal a low frequency of responders to PvCelTOS, since only 17.8% of the studied population (94 individuals) presented specific IgG antibodies against the protein. Interestingly, the epidemiological data were similar between responders and NRs against this protein (Table 1). On both groups, responders and NRs, the age, time of residence in endemic area, the number of previous malaria episodes, the number of recent malaria episodes, the frequency of individuals with recent malaria episodes, and months elapsed from the last malaria episode were similar (*p* > 0.05).

**Figure 1:**
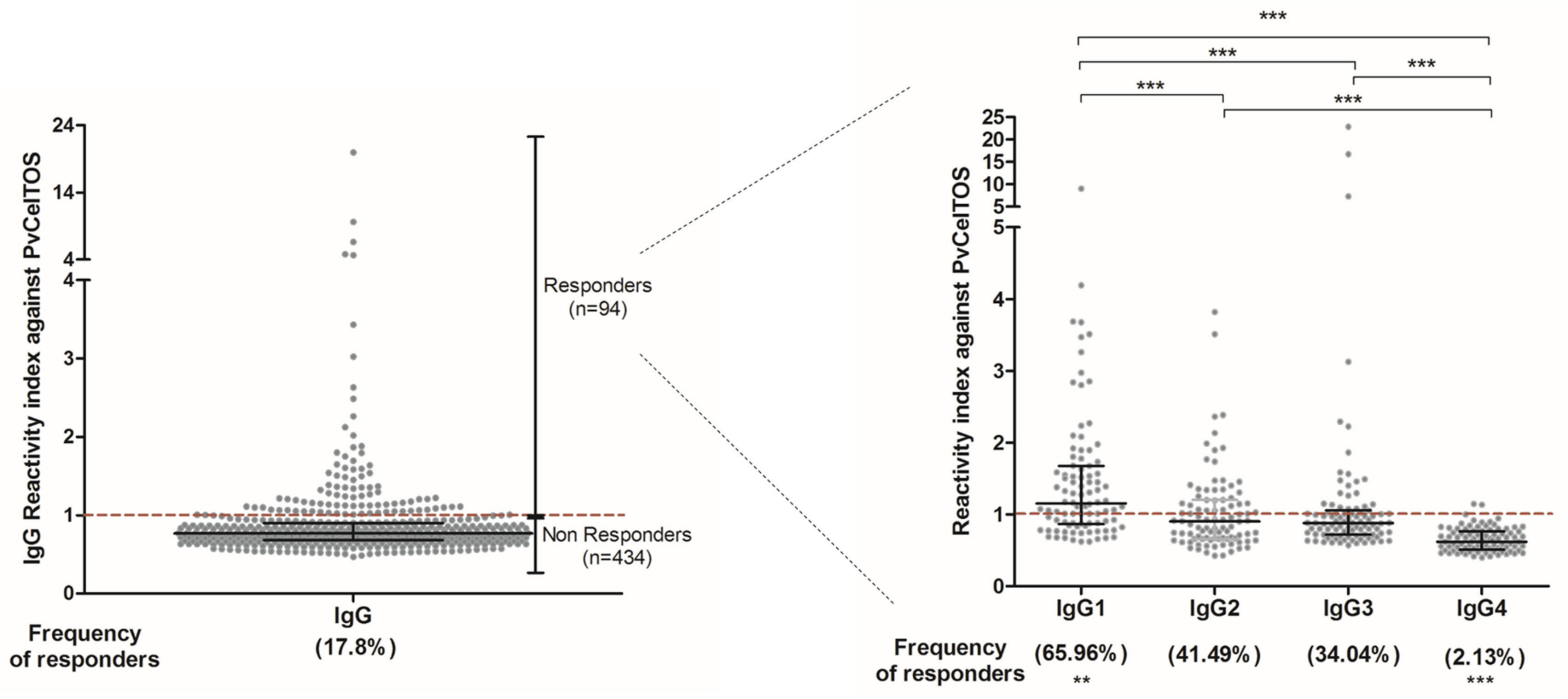
**Reactivity index (RI) of IgG and subclass against PvCelTOS**. On both graphs, each point represents an individual RI against PvCelTOS and the red traced line represents the cutoff. Ninety-four individuals presented RI against PvCelTOS higher than 1 and were considered responders to this protein. Among the responders, IgG1 was the prevalent subclass in comparison to IgG2 (*p* = 0.0012), IgG3 (*p* < 0.0001), and IgG4 (*p* < 0.0001). Additionally, the RI of IgG1 was higher than the RI of all other subclasses (*p* < 0.0001), and the RI of IgG4 was statistically lower than all other subclasses (*p* < 0.0001).

Among the group of responders to PvCelTOS, the RI ranged from 1.01 to 19.93 (median = 1.205; interquartile range = 1.082; 1.552), reflecting a wide spectrum in magnitude of naturally acquired IgG response. The IgG subclass profile was marked by IgG1, the most prevalent subclass, present in 65.96% of responders, and with major RI (median = 1.15; interquartile range = 0.86–1.68) compared to IgG2 (median = 0.9; interquartile range = 0.66–1.2), IgG3 (median = 0.88; interquartile range = 0.72–1.06), and IgG4 (median = 0.62; interquartile range = 0.51–0.76) (Figure 1). Moreover, IgG3 RIs were directly correlated to the number of recent malaria episodes (*p* = 0.003; *r* = 0.315; Figure S1A in Supplementary Material) and inversely associated with the time elapsed from the last malaria episode (*p* = 0.031; *r* = −0.258; Figure S1B in Supplementary Material).

### High IgG RIs against PvCelTOS Are Driven by Cytophilic Antibodies and Associated with Recent Infections

**Figure 2:**
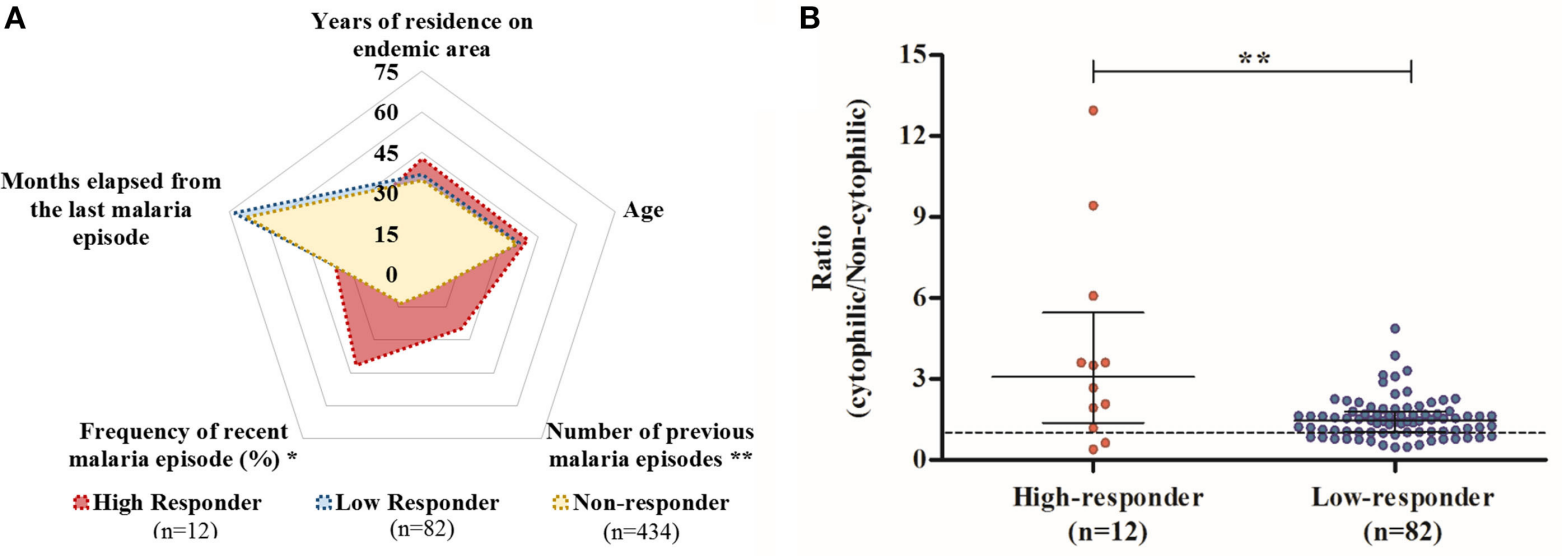
**Comparison of profiles of high responders (HRs), low responders (LRs), and non-responders (NRs) to PvCelTOS**. **(A)** Diagram of epidemiological data. The mean values of epidemiological parameters of HRs, LRs, and NRs to PvCelTOS are represented by red, blue, and yellow areas, respectively. Age and time of residence in endemic areas are expressed in years, while time elapsed from the last malaria episode is expressed in months and frequency of recent malaria episodes indicates the percentage of individuals who reported malaria episodes in the last year. HRs presented a higher number of previous malaria episodes and higher frequency of recent malaria episodes than LRs (*p* = 0.0051 and *p* = 0.02, respectively) **(B)** Comparison of ratio (cytophilic/non-cytophilic) antibodies. Red and blue points represent the HR and LR, respectively. Points above the value of 1 represent individuals with a cytophilic profile of IgG (IgG1 + IgG3, higher than IgG2 + IgG4).

In order to identify possible factors that could be associated with this large spectrum of reactivity against PvCelTOS in IgG-positive individuals, we explored epidemiological data among responders. Initially, we observed that the RI against PvCelTOS was directly correlated with the number of previous malaria episodes (*p* = 0.047; *r* = 0.227; Figure S1C in Supplementary Material) and inversely correlated with the time elapsed from the last malaria episode (*p* = 0.045; *r* = −0.24; Figure S1D in Supplementary Material). Based on these findings, responder individuals were divided into two subgroups: high responders (HRs; individuals who had RI of IgG against PvCelTOS higher than 2) and low responders (LRs; individuals who had RI of IgG against PvCelTOS between 1 and 2). Figure 2A illustrates the means of epidemiological parameters of HRs, LRs, and NRs to PvCelTOS. Interestingly, while NRs and LRs presented a very similar profile of epidemiological parameters, HRs presented a statistically higher number of previous malaria episodes in comparison to NR and LR (*p* = 0.0058; *p* = 0.0051, respectively). Moreover, despite no statistical differences could be observed on the time elapsed from the last malaria episode (*p* = 0.15 in ANOVA test), the frequency of individuals who reported recent episodes of malaria was higher in HR (41.6%) than LR (12%, *p* = 0.02) and NR (13.1%, *p* = 0.016). Moreover, the proportion of RIs of cytophilic over non-cytophilic antibodies (IgG1 + IgG3/IgG2 + IgG4) presented direct correlation with RI of IgG of responder individuals (*p* = 0.0016; *r* = 0.32), suggesting that higher RI could be associated with a cytophilic profile of humoral response against PvCelTOS. Interestingly, although the proportion of individuals with cytophilic profile was similar in both groups, HR and LR (83% and 78%, respectively), the ratio of (cytophilic/non-cytophilic) antibodies was significantly higher in HR than LR (*p* = 0.0076) (Figure 2B).

### Five Immunogenic Regions Identified in PvCelTOS and Two Linear B-Cell Epitopes Broadly Recognized by Naturally Acquired IgG Antibodies

Four B-cell linear epitopes were predicted *in silico* in the entire sequence of PvCelTOS (PvCelTOS_K6-N13_; PvCelTOS_G38-R57_; PvCelTOS_I136-E143_; PvCelTOS_K166-S191_).

**Figure 3:**
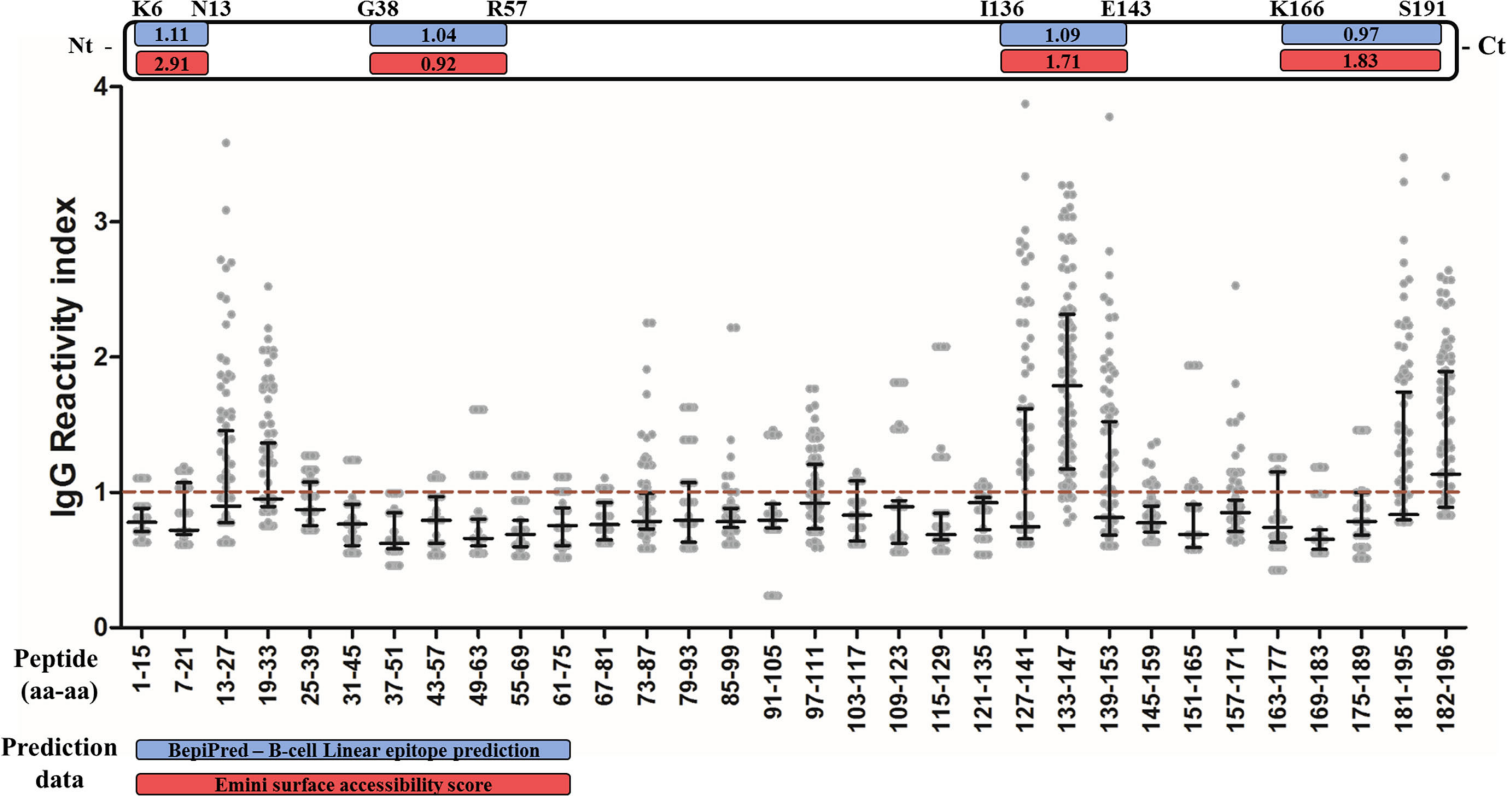
**Mapping of B-cell epitopes in PvCelTOS**. Each column represents a peptide, the numbers indicate the first and last amino acid (aa-aa) of the peptide. The points represent the value of IgG reactivity index (RI) specific for each peptide of one responder to PvCelTOS and the red traced line represents the cutoff value. The black lines indicate median and interquartile range. If the RI for one peptide was higher than 1, the individual was considered positive to this peptide. The white bar on top represents the linear structure of the protein, in which the blue boxes indicate the BepiPred prediction score and red boxes indicate the Emini surface accessibility score of predicted linear epitopes.

In order to validate the prediction data and identify possible non-predicted immunogenic regions of PvCelTOS, plasma from IgG responders to PvCelTOS was tested against 32 overlapping peptides corresponding to the complete amino acid sequence. First, 10 peptides (N13-L27; S19-V33; E73-I87; L79-K93; S97-A111; P127-V141; I133-G147; P139-V153; L181-L195; E182-D196) were broadly recognized by responders to PvCelTOS (Figure 3). Two of the predicted epitopes (PvCelTOS_I136-E143_ and PvCelTOS_K166-S191_) were present (partially or entirely) in peptides confirmed as naturally immunogenic. Interestingly, peptides I133-G147 and E182-D196 were recognized by IgG specific antibodies of responders to PvCelTOS in frequencies higher than 50% (92% and 54%, respectively) and presented median of RI higher than 1 (1.79 and 1.14, respectively). In addition, peptides P127-V141, P139-V153, and L181-L195 were located besides the most immunogenic peptides and presented overlapped sequences, which were also recognized by IgG antibodies in moderate frequencies. Peptide I133-G147 (ASTIKPPRVSEDAYF) presented the highest IgG RI (*p* < 0.0001 by ANOVA test) and the highest frequency of recognition (92%) compared to all other peptides. While it contains the entire sequence of predicted epitope PvCelTOS_136-143_, peptides P127-V141 and P139-V153, which contain only the partial sequence of the predicted epitope, presented minor frequencies of recognition (38% and 39%, respectively; *p* < 0.0001 on Fisher’s exact test). The peptides L181-L195 and 186-196 were both partially inserted in the predicted linear epitope PvCelTOS_166-191_ and could be the immune dominant sequence of this longer predicted epitope. These data supported the prediction of linear B-cell epitopes PvCelTOS_I136-E146_ and PvCelTOSK_166-S191_. Conversely, peptides N13-L27, S19-V33, and S97-A111 also presented frequency of recognition about 40% (38, 40, and 36%, respectively). After the confirmation of five immunogenic regions and two immunodominant epitopes in PvCelTOS, we also compared the RI and frequencies between HR and LR for PvCelTOS. However, no differences were found.

### Main B Cell Epitopes Are Present on PvCelTOS Surface

**Figure 4:**
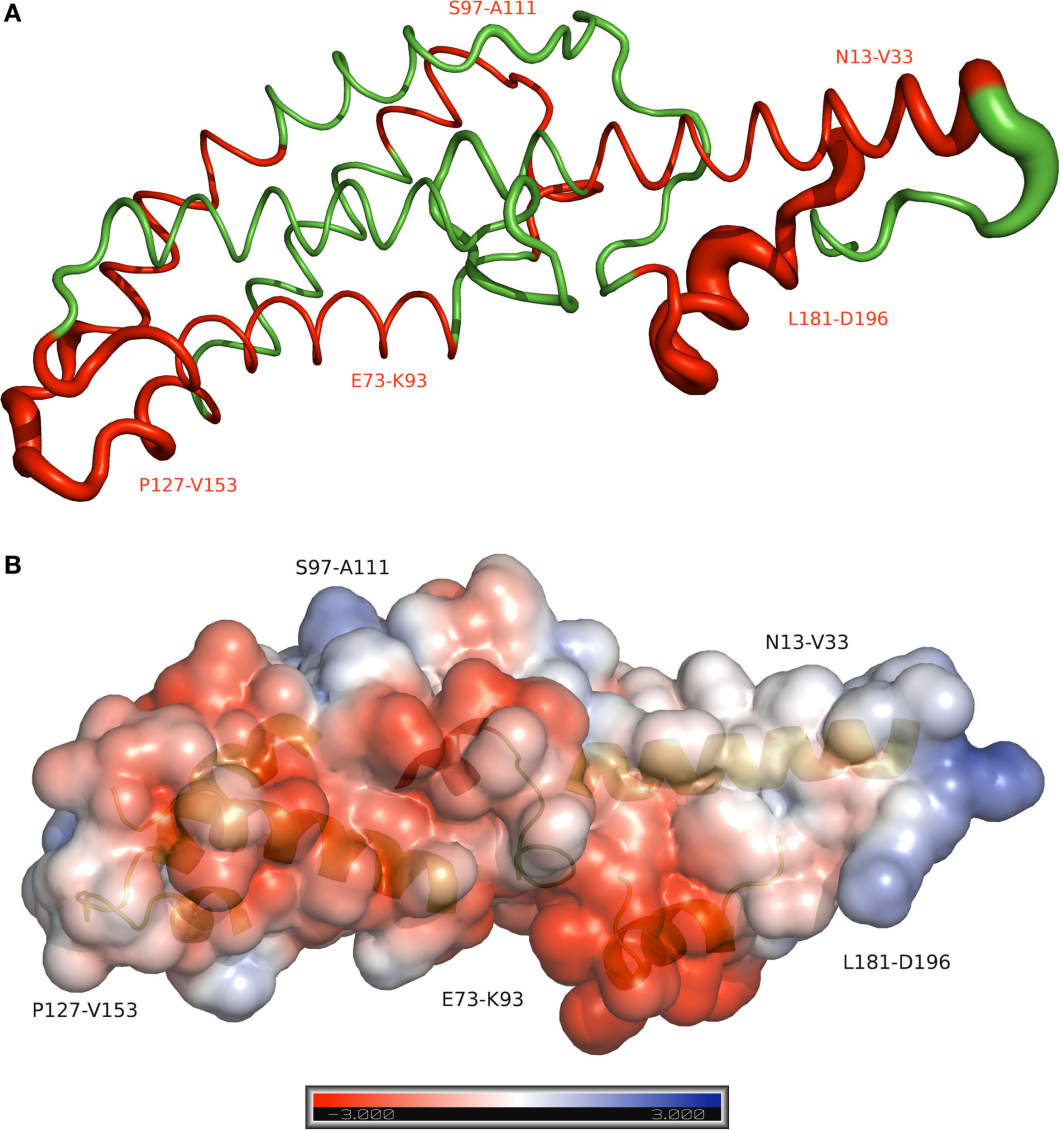
**Molecular dynamics and electrostatic potential surface for the PvCelTOS**. **(A)** Sausage plot of the PvCelTOS. The red color identifies the immunogenic regions of PvCelTOS. Thickness depicts relative fluctuation as calculated during molecular dynamics. The thinnest segments represent the most stable regions of the protein. **(B)** The surface model shows the electrostatic potential surface of the PvCelTOS, representing the positive (blue) and negative (red) charges. The secondary structure in the background represents the immunogenic region.

Peptides that presented overlapped amino acids and were recognized by more than 20% of responders to PvCelTOS (Figure 3) were grouped as immunogenic regions. All peptides inserted in identified immunogenic regions are listed in Table 2 with their respective frequencies of recognition, BepiPred and ESA scores. In this context, we identified five immunogenic regions PvCelTOS_N13-V33_, PvCelTOS_E73-K93_, PvCelTOS_S97-A111_, PvCelTOS_P127-V153_, and PvCelTOS_L181-D196_, in which B-cell epitopes could be inserted. Interestingly, the peptides with higher frequency of specific responders (I133-G147, L181-L195, and 182-186) presented a good combination of BepiPred and ESA score. The molecular dynamics and electrostatic potential surface of PvCelTOS indicate regions P127-V153, N13-V33, and L181-D186 as more flexible than E73-K93 and S97-A111 (Figure 4A). Regarding solvent exposure, all immunogenic regions were exposed and accessible in solution. Interestingly, the immunogenic regions L181-D196 and E73-K93 are part of a very negatively charged region, while N13-V33 and P127-V153 are in a mostly neutral-positive region (Figure 4B).

**Table 2:** **Identification of immunogenic regions in PvCelTOS**.

Immunogenic region	Sequence	Reactivity index (RI)Mean (CI 95%)	Responders (%)	Position	Peptide sequence	Specific responders (%)	BepiPred score	ESA score
PvCelTOS_N13-V33_	NKVNRV**SIICAFLAL**FCFVNV	1.17 (1.07–1.27)	45%	13–27	NKVNRV**SIICAFLAL**	38%	−1.52	0.35
				19–33	**SIICAFLAL**FCFVNV	40%	−2.37	0.07
PvCelTOS_E73-K93_	EVIGNE**LADNIANEI**VSSLQK	0.93 (0.88–0.99)	30%	73–87	EVIGNELADNIANEI	22%	0.01	0.70
				79–93	LADNIANEIVSSLQK	20%	0.14	0.56
PvCelTOS_S97-A111_	SFLQSGFDVKTQLKA	0.95 (0.89–1.01)	36%	97–111	SFLQSGFDVKTQLKA	36%	0.12	0.91
PvCelTOS_P127-V153_	PTEKIVAST**IKPPRVSEDAYFLLG**PVV	1.39 (1.27–1.50)	69%	127–141	PTEKIVASTIKPPRV	38%	0.69	0.89
				133–147	IKPPRVSEDAYFLLG	92%	0.52	1.14
				139–153	PRVSEDAYFLLGPVV	39%	−0.08	0.75
PvCelTOS_L181-D196_	L**EEEEAEDEFSDELL**D	1.32 (1.21–1.44)	53%	181–195	LEEEEAEDEFSDELL	43%	0.95	2.15
				182–196	EEEEAEDEFSDELLD	54%	0.84	2.08

*Peptides with overlapping and recognized by more than 20% of responders to PvCelTOS were grouped in immunogenic regions. The RI of an immunogenic region represents the mean of RI of all peptides inserted in that immunogenic region with a confident interval of 95%. The frequency of recognition of immunogenic regions was defined based on the number of individuals whith RI to immunogenic region higher than 1. The peptides combined in an immunogenic region were listed with their respective frequencies of recognition, BepiPred score, and Emini surface accessibility (ESA) score. Overlapped mers were signalized by underlined bold typeface on immunogenic region sequence*.

## Discussion

Despite significant advances in the understanding of the biology of *Plasmodium* parasites and the immune response elicited by these pathogens, there is not yet a subunit vaccine capable of providing long-lasting protection. The cell-traversal protein for ookinetes and sporozoites (CelTOS) has been considered a potential novel alternative for a vaccine against malaria (29, 32, 41), but the knowledge on *P. vivax* CelTOS potential remains scarce. Unfortunately, many conventional vaccinology strategies applied to *P. falciparum* are especially difficult when dealing with non-cultivable microorganisms such as *P. vivax*. Consequently, seroepidemiological studies have played a significant role in the identification and validation of *P. vivax* vaccine candidates (42–48). Therefore, we confirmed the naturally acquired humoral response against PvCelTOS (IgG and IgG subclass) and identified five B-cell epitopes along the entire PvCelTOS amino acid sequence, which were recognized by IgG antibodies from malaria-exposed populations from Brazilian Amazon.

Plasma samples were collected in three cross-sectional studies with Brazilian Amazon communities between 2013 and 2015. The profile of the studied individuals shows that our population included rainforest region natives and migrants from non-endemic areas of Brazil who had lived in the area for more than 10 years. The majority of individuals reported a prior experience with *P. vivax* and/or *P. falciparum* malaria. Concerning malaria history, the highly variable range of number of previous infections, time of residence in endemic areas, and time since the last infection suggests differences in exposure and immunity, since it is well known that the acquisition of clinical immunity mediated by antibodies depends on continued exposure to the parasite (49–51). The correlation between time of residence in endemic areas and months since the last infection observed in our study also indicates that this phenomenon could be occurring in low/medium endemic areas like the Brazilian Amazon. Therefore, the selection of these individuals was ideal to detect the presence of antibodies against the new recombinant antigen and distinguish whether the alterations found were related to malaria exposure and/or indicatives of protection.

First, we found 94 individuals presenting specific antibodies to PvCelTOS and confirmed the natural immunogenicity of PvCelTOS among exposed individuals from Brazilian Amazon. Recently, Longley and collaborators also reported the first evidence of naturally induced IgG responses to *Pv*CelTOS in human volunteers from Western Thailand (33). Interestingly, the frequency of responders to PvCelTOS observed in our studied population (17.8%) was similar to the frequency observed by Longley on uninfected and clinical malaria individuals (33). Moreover, the low humoral reactivity against PvCelTOS is commonly found in other *Plasmodium* preerythrocytic antigens (48, 52, 53). The short life of specific antibodies, host genetic factors, and/or epidemiological parameters could be possible reasons for the low frequency of responders against PvCelTOS in endemic areas. The short life of specific PvCelTOS humoral response hypothesis does not seem to occur since Longley et al. verified that IgG positivity and magnitude of response were present over the 1-year period in the absence of *P. vivax* infections (33). Our study also describes anti-PvCelTOS antibodies in individuals who reported no malaria in the last 10 years or more. However, in both cases, the contact between human host and sporozoite antigens in transmission areas was not evaluated. In relation to host genetic factors, there is a significant body of evidences of its influence in malaria outcomes and the capacity to mount a humoral immune response (54–57). To date, associations of HLA class II on humoral immune response to malaria antigens were reported in individuals living in malaria-endemic areas from Brazilian Amazon (58, 59) and in human vaccine trials (60–62). In *P. vivax* preerythrocytic targets, the presence of HLA-DRB1*03 and DR5 was associated with the absence of antibody response to the CSP amino-terminal region (48) and HLA-DRB1*07 was related to the absence of specific antibodies for CSP repeats of VK210 (52). Moreover, Chaves and collaborators reported that PvCelTOS gene sequence is highly conserved among isolates from different Brazilian geographic regions (unpublished data), suggesting a low selective pressure by immune response against PvCelTOS. In our view, the influence of immunogenetic factors in PvCelTOS-specific humoral response are feasible, but more studies are still necessary to confirm this hypothesis.

Regarding the influence of epidemiological factors, we initially tried to investigate the associations between exposition to malaria and the frequency of IgG responders to PvCelTOS. Surprisingly, although the association of epidemiological data with specific response against *Plasmodium* antigens was well characterized on several studies (63–65), we observed a similar epidemiological profile between responders and NRs to PvCelTOS. Therefore, we focused on the search of distinct epidemiological and IgG subclass profiles among PvCelTOS responder individuals. The knowledge about the antibody subclass profile is critical to suggest functional antimalarial immunity and to evaluate potential vaccine candidates. Cytophilic antibodies (IgG1 and IgG3) are frequently prevalent on immune serum from high-transmission areas (66–69) and often correlate with protection from disease (70–72). In our study, IgG1 presented higher frequencies of responders and median RI than all other subclasses. Moreover, IgG3 RIs were directly associated with the number of malaria episodes over the last 12 months and inversely correlated with the time elapsed from the last malaria episode, suggesting that recent *P. vivax* infections can raise the levels of anti-PvCelTOS specific IgG3. The sterile protective immunity to malaria was recently associated with a panel of antigens (28), and the relationship of cytophilic antibodies and reduced risk of symptoms are a common finding in high endemic areas (70–74). However, in our study, concerning the higher levels of IgG1 for PvCelTOS and the association of IgG3 levels with recent infections, we cannot confirm or discard its role as part of protective humoral response until more conclusive studies, such as sporozoite inhibition by anti-PvCelTOS specific antibodies, are conducted. In the same way, among responders, IgG RIs were directly correlated with the number of previous malaria episodes and inversely correlated with the time elapsed from the last malaria episodes, suggesting that antibody levels for PvCelTOS could be associated with recent infections.

The influence of epidemiological parameters on immunity to malaria was previously observed in studies from Brazilian Amazon population. Based on previous studies that associated high levels of antibodies with multiple preerythrocytic antigens with reduced risk of clinical malaria in children (75) and decreased risk of infection in adults (68), we also aimed to investigate if the epidemiological parameters could reveal new findings about the role of exposition on PvCelTOS immunogenicity. Therefore, we subdivided the large spectrum IgG RIs among PvCelTOS responders into HRs (RI > 2) and LRs (RI < 2). Although LRs and NRs to PvCelTOS presented similar exposition factors to malaria, interestingly, HR individuals presented a remarkable higher number of previous malaria episodes, frequency of recent malaria episodes, and a higher ratio of cytophilic/non-cytophilic antibodies than LRs. This observation suggested that higher level of exposition to malaria induced a more intense and improved humoral response against PvCelTOS. Unfortunately, the cross-sectional design of our study limited the investigation to retrospective malaria histories, and the best approximation of an individual’s protection was the estimated amount of time that had passed since their last malaria episode, which presented no significant association with IgG response against PvCelTOS. Prospective studies on humoral immune responses and studies addressing the ability of these antibodies to interfere the motility/invasion of sporozoites (76, 77) will provide more evidences of the protective role of anti-PvCelTOS antibodies.

Information at the amino acid level about the epitopes of proteins recognized by antibodies is important for their use as biological tools and for understanding general molecular recognition events (78). In this context, epitope prediction programs have been widely used in malaria research (4, 79–81). Nevertheless, the use of chemically prepared arrays of short peptides is a more powerful tool to identify and characterize epitopes recognized by antibodies (46, 82, 83). It is also important to mention that in order to raise antibodies for a peptide, a minimum length of six amino acids is required, and peptides of >10 amino acids are generally required for the induction of antibodies that may bind to the native protein (84). In this context, the synthesis of 15 amino acid peptides, with 9 overlapping, has allowed the identification of PvCelTOS B-cell epitopes encompassed in sequences ranging from 15 to 27 amino acids in length. Therefore, after the confirmation of PvCelTOS as naturally immunogenic in exposed populations, the present paper describes for the first time the fine B cell epitope mapping of a full-length protein. Initially, 10 peptides were specifically recognized by naturally acquired antibodies from PvCelTOS responders. After a combination of *in silico* approaches and recognition of overlapped peptides, five immunogenic regions were confirmed (PvCelTOS_13-33_, PvCelTOS_73-93_, PvCelTOS_97-111_, PvCelTOS_127-153_, and PvCelTOS_181-196_) in different frequencies and RIs. Moreover, the main linear epitope (ASTIKPPRVSEDAYF) presented highest IgG RI and frequency compared to all other naturally recognized peptides, suggesting that the majority of naturally acquired antibodies against PvCelTOS are directed to the C-terminal region. Moreover, T cell responses to PvCelTOS may also help to determine the immunodominant repertoire in individuals living in malaria-endemic regions, which could also supply information for the development of a vaccine for PvCelTOS. In humans, PfCelTOS derivate peptides elicited proliferative and IFN-γ responses in *ex vivo* ELISPOT assays using peripheral blood mononuclear cells from naturally exposed individuals living in Ghana (30).

Recently, CelTOS was demonstrated as highly conserved protein across several large groups of apicomplexan parasites including *Plasmodium* spp., *Cytauxzoon, Theileria*, and *Babesia* and considered essential to cell infection, traversal, and membrane disruption (85). Despite the genetical differences between PfCelTOS and PvCelTOS, it is important to mention that Bergmann-Leitner and colleagues immunized mice and rabbits with recombinant PfCelTOS and also observed specific antibodies for linear B-cell epitopes at C-terminal (82). These observations suggested that CelTOS could present a similar conformation among species, with similar regions targeted by antibodies. We considered that the exposition of linear epitopes is a critical step to their recognition by circulating antibodies; therefore, the combination of ESA, molecular dynamics, and electrostatic potential surface was used as a complementary approach to predict the exposition of epitope sequences on protein surface. All immunogenic regions identified were exposed and accessible to antibodies. This finding could be important in a future subunit vaccine composition based on these identified regions. However, the potential of these specific antibodies directed main PvCelTOS epitopes in the inhibition of sporozoite motility, invasion, and/or traversal remains to be investigated.

## Author Contributions

JL-J did study designing, performed experiments, data analysis, manuscript preparation, and manuscript review. RR-d-S did study designing, performed experiments, data analysis, and manuscript preparation. IS performed experiments. CL-C did recombinant protein expression and manuscript review. JM did molecular dynamics and bioinformatics and manuscript review. DP-d-S performed collection of blood and epidemiological data. AF did fieldwork support. AT performed collection of blood and epidemiological data and diagnosis. FP did fieldwork support. LC performed experiments. LP-R did data analysis and manuscript review. AR-S did recombinant protein expression, data analysis, and manuscript review. DB did study designing, fieldwork support, manuscript review, and data analysis.

## Conflict of Interest Statement

The authors declare that the research was conducted in the absence of any commercial or financial relationships that could be construed as a potential conflict of interest.

## Supplementary Material

Figure S1 **Associations of humoral response and exposition parameters in responders to PvCelTOS**. **(A)** Spearman correlation between IgG3 reactivity index (RI) and number of recent malaria episodes; **(B)** Spearman correlation between IgG3 and months elapsed since the last malaria episode; **(C)** Spearman correlation between anti-PvCelTOS IgG reactivity index and number of previous malaria episodes; and **(D)** Spearman correlation between anti-PvCelTOS IgG RI and months elapsed since the last malaria episode.

## References

[1] W.H.O. World Malaria Report. Geneva: WHO (2015).

[2] Richie TL Saul A. Progress and challenges for malaria vaccines. Nature (2002) 415(6872):694–701. 10.1038/415694a 11832958

[3] W.H.O. World Malaria Report 2014. Geneva: WHO (2014).

[4] Rodrigues-da-Silva RN Martins da Silva JH Singh B Jiang J Meyer EV Santos F In silico identification and validation of a linear and naturally immunogenic B-cell epitope of the *Plasmodium vivax* malaria vaccine candidate merozoite surface protein-9. PLoS One (2016) 11(1):e0146951. 10.1371/journal.pone.0146951 26788998 PMC4720479

[5] WHO. Tables of Malaria Vaccine Projects Globally ("Rainbow Tables"). (2015). Available from: http://www.who.int/immunization/research/development/Rainbow_tables/en/

[6] Tan LK Yacoub S Scott S Bhagani S Jacobs M. Acute lung injury and other serious complications of *Plasmodium vivax* malaria. Lancet Infect Dis (2008) 8(7):449–54. 10.1016/S1473-3099(08)70153-1 18582837

[7] Price RN Tjitra E Guerra CA Yeung S White NJ Anstey NM Vivax malaria: neglected and not benign. Am J Trop Med Hyg (2007) 77(6 Suppl):79–87. 18165478 PMC2653940

[8] Rahimi BA Thakkinstian A White NJ Sirivichayakul C Dondorp AM Chokejindachai W. Severe vivax malaria: a systematic review and meta-analysis of clinical studies since 1900. Malar J (2014) 13:481. 10.1186/1475-2875-13-481 25486908 PMC4364574

[9] O’Brien AT Ramirez JF Martinez SP A descriptive study of 16 severe *Plasmodium vivax* cases from three municipalities of Colombia between 2009 and 2013. Malar J (2014) 13:404 10.1186/1475-2875-13-404 25318617 PMC4203896

[10] Gougoutsi A Karageorgopoulos DE Dimitriadou A Melas N Kranidiotis G Voutsinas D Severe *Plasmodium vivax* malaria complicated with acute respiratory distress syndrome: a case associated with focal autochthonous transmission in Greece. Vector Borne Zoonotic Dis (2014) 14(5):378–81. 10.1089/vbz.2012.1192 24745658

[11] Zubairi AB Nizami S Raza A Mehraj V Rasheed AF Ghanchi NK Severe *Plasmodium vivax* malaria in Pakistan. Emerg Infect Dis (2013) 19(11):1851–4. 10.3201/eid1911.130495 24188313 PMC3837647

[12] Price RN von Seidlein L Valecha N Nosten F Baird JK White NJ. Global extent of chloroquine-resistant *Plasmodium vivax*: a systematic review and meta-analysis. Lancet Infect Dis (2014) 14(10):982–91. 10.1016/S1473-3099(14)70855-2 25213732 PMC4178238

[13] de Santana Filho FS Arcanjo AR Chehuan YM Costa MR Martinez-Espinosa FE Vieira JL Chloroquine-resistant *Plasmodium vivax*, Brazilian Amazon. Emerg Infect Dis (2007) 13(7):1125–6. 10.3201/eid1307.061386 18214203 PMC2878224

[14] Ruebush TK II Zegarra J Cairo J Andersen EM Green M Pillai DR Chloroquine-resistant *Plasmodium vivax* malaria in Peru. Am J Trop Med Hyg (2003) 69(5):548–52. 14695094

[15] Nayar JK Baker RH Knight JW Sullivan JS Morris CL Richardson BB Studies on a primaquine-tolerant strain of *Plasmodium vivax* from Brazil in Aotus and Saimiri monkeys. J Parasitol (1997) 83(4):739–45. 10.2307/3284254 9267419

[16] Kristensen KL Dragsted UB. Recurrent *Plasmodium vivax* malaria due to dose-dependent primaquine resistance: a case report. Scand J Infect Dis (2014) 46(1):63–5. 10.3109/00365548.2013.822093 23957539

[17] Arias AE Corredor A Low response of Colombian strains of *Plasmodium vivax* to classical antimalarial therapy. Trop Med Parasitol (1989) 40(1):21–3. 2662351

[18] Swearingen KE Lindner SE Shi L Shears MJ Harupa A Hopp CS Interrogating the Plasmodium Sporozoite Surface: Identification of Surface-Exposed Proteins and Demonstration of Glycosylation on CSP and TRAP by Mass Spectrometry-Based Proteomics. PLoS Pathog (2016) 12(4):e1005606. 10.1371/journal.ppat.1005606 27128092 PMC4851412

[19] Clyde DF McCarthy VC Miller RM Hornick RB Specificity of protection of man immunized against sporozoite-induced falciparum malaria. Am J Med Sci (1973) 266(6):398–403. 10.1097/00000441-197309000-00002 4590095

[20] Rieckmann KH Carson PE Beaudoin RL Cassells JS Sell KW Letter: sporozoite induced immunity in man against an Ethiopian strain of *Plasmodium falciparum*. Trans R Soc Trop Med Hyg (1974) 68(3):258–9. 10.1016/0035-9203(74)90129-1 4608063

[21] Egan JE Hoffman SL Haynes JD Sadoff JC Schneider I Grau GE Humoral immune responses in volunteers immunized with irradiated *Plasmodium falciparum* sporozoites. Am J Trop Med Hyg (1993) 49(2):166–73. 8357078 10.4269/ajtmh.1993.49.166

[22] Hoffman SL Goh LM Luke TC Schneider I Le TP Doolan DL Protection of humans against malaria by immunization with radiation-attenuated *Plasmodium falciparum* sporozoites. J Infect Dis (2002) 185(8):1155–64. 10.1086/339409 11930326

[23] Olotu A Fegan G Wambua J Nyangweso G Awuondo KO Leach A Four-year efficacy of RTS,S/AS01E and its interaction with malaria exposure. N Engl J Med (2013) 368(12):1111–20. 10.1056/NEJMoa1207564 23514288 PMC5156295

[24] Rts SCTP Agnandji ST Lell B Fernandes JF Abossolo BP Methogo BG A phase 3 trial of RTS,S/AS01 malaria vaccine in African infants. N Engl J Med (2012) 367(24):2284–95. 10.1056/NEJMoa1208394 23136909 PMC10915853

[25] Bejon P Cook J Bergmann-Leitner E Olotu A Lusingu J Mwacharo J Effect of the pre-erythrocytic candidate malaria vaccine RTS,S/AS01E on blood stage immunity in young children. J Infect Dis (2011) 204(1):9–18. 10.1093/infdis/jir222 21628653 PMC3105039

[26] Olotu A Lusingu J Leach A Lievens M Vekemans J Msham S Efficacy of RTS,S/AS01E malaria vaccine and exploratory analysis on anti-circumsporozoite antibody titres and protection in children aged 5-17 months in Kenya and Tanzania: a randomised controlled trial. Lancet Infect Dis (2011) 11(2):102–9. 10.1016/S1473-3099(10)70262-0 21237715 PMC3341451

[27] Gruner AC Mauduit M Tewari R Romero JF Depinay N Kayibanda M Sterile protection against malaria is independent of immune responses to the circumsporozoite protein. PLoS One (2007) 2(12):e1371. 10.1371/journal.pone.0001371 18159254 PMC2147056

[28] Trieu A Kayala MA Burk C Molina DM Freilich DA Richie TL Sterile protective immunity to malaria is associated with a panel of novel *P. falciparum* antigens. Mol Cell Proteomics (2011) 10(9):M111007948. 10.1074/mcp.M111.007948 21628511 PMC3186199

[29] Kariu T Ishino T Yano K Chinzei Y Yuda M. CelTOS, a novel malarial protein that mediates transmission to mosquito and vertebrate hosts. Mol Microbiol (2006) 59(5):1369–79. 10.1111/j.1365-2958.2005.05024.x 16468982

[30] Anum D Kusi KA Ganeshan H Hollingdale MR Ofori MF Koram KA Measuring naturally acquired ex vivo IFN-gamma responses to *Plasmodium falciparum* cell-traversal protein for ookinetes and sporozoites (CelTOS) in Ghanaian adults. Malar J (2015) 14:20 10.1186/s12936-014-0539-5 25604473 PMC4308902

[31] Kusi KA Bosomprah S Dodoo D Kyei-Baafour E Dickson EK Mensah D Anti-sporozoite antibodies as alternative markers for malaria transmission intensity estimation. Malar J (2014) 13:103. 10.1186/1475-2875-13-103 24635830 PMC3995447

[32] Bergmann-Leitner ES Mease RM De La Vega P Savranskaya T Polhemus M Ockenhouse C Immunization with pre-erythrocytic antigen CelTOS from *Plasmodium falciparum* elicits cross-species protection against heterologous challenge with *Plasmodium berghei*. PLoS One (2010) 5(8):e12294. 10.1371/journal.pone.0012294 20808868 PMC2924390

[33] Longley RJ Reyes-Sandoval A Montoya-Diaz E Dunachie S Kumpitak C Nguitragool W Acquisition and longevity of antibodies to *Plasmodium vivax* preerythrocytic antigens in Western Thailand. Clin Vaccine Immunol (2016) 23(2):117–24. 10.1128/CVI.00501-15 PMC4744911 26656115

[34] Stanisic DI Fowkes FJ Koinari M Javati S Lin E Kiniboro B Acquisition of antibodies against *Plasmodium falciparum* merozoites and malaria immunity in young children and the influence of age, force of infection, and magnitude of response. Infect Immun (2015) 83(2):646–60. 10.1128/IAI.02398-14 25422270 PMC4294228

[35] Larsen JE Lund O Nielsen M Improved method for predicting linear B-cell epitopes. Immunome Res (2006) 2:2 10.1186/1745-7580-2-2 16635264 PMC1479323

[36] Emini EA Hughes JV Perlow DS Boger J. Induction of hepatitis A virus-neutralizing antibody by a virus-specific synthetic peptide. J Virol (1985) 55(3):836–9. 2991600 10.1128/jvi.55.3.836-839.1985 PMC255070

[37] Berendsen HJC van der Spoel D van Drunen R GROMACS: a message-passing parallel molecular dynamics implementation. Comput Phys Commun (1995) 91(1–3):13 10.1016/0010-4655(95)00042-E

[38] Oostenbrink C Villa A Mark AE van Gunsteren WF. A biomolecular force field based on the free enthalpy of hydration and solvation: the GROMOS force-field parameter sets 53A5 and 53A6. J Comput Chem (2004) 25(13):1656–76. 10.1002/jcc.20090 15264259

[39] William LJ Chandrasekhar J Madura JD Impey RW Klein ML Comparison of simple potential functions for simulating liquid water. J Chem Phys (1983) 79:926–35. 10.1063/1.445869

[40] Baker NA Sept D Joseph S Holst MJ McCammon JA. Electrostatics of nanosystems: application to microtubules and the ribosome. Proc Natl Acad Sci U S A (2001) 98(18):10037–41. 10.1073/pnas.181342398 11517324 PMC56910

[41] Bergmann-Leitner ES Legler PM Savranskaya T Ockenhouse CF Angov E. Cellular and humoral immune effector mechanisms required for sterile protection against sporozoite challenge induced with the novel malaria vaccine candidate CelTOS. Vaccine (2011) 29(35):5940–9. 10.1016/j.vaccine.2011.06.053 21722682

[42] Bueno LL Morais CG Soares IS Bouillet LE Bruna-Romero O Fontes CJ *Plasmodium vivax* recombinant vaccine candidate AMA-1 plays an important role in adaptive immune response eliciting differentiation of dendritic cells. Vaccine (2009) 27(41):5581–8. 10.1016/j.vaccine.2009.07.031 19651176

[43] Amarasinghe S Kathriarachchi H Udagama P. Conserved regions of *Plasmodium vivax* potential vaccine candidate antigens in Sri Lanka: conscious in silico analysis of prospective conformational epitope regions. Asian Pac J Trop Med (2014) 7(10):832–40. 10.1016/S1995-7645(14)60146-2 25129470

[44] Xia H Fang Q Jangpatarapongsa K Zhiyong T Cui L Li B A comparative study of natural immune responses against *Plasmodium vivax* C-terminal merozoite surface protein-1 (PvMSP-1) and apical membrane antigen-1 (PvAMA-1) in two endemic settings. EXCLI J (2015) 14:926–34. 10.17179/excli2015-388 26713085 PMC4677636

[45] Lima-Junior JC Tran TM Meyer EV Singh B De-Simone SG Santos F Naturally acquired humoral and cellular immune responses to *Plasmodium vivax* merozoite surface protein 9 in Northwestern Amazon individuals. Vaccine (2008) 26(51):6645–54. 10.1016/j.vaccine.2008.09.029 18832003 PMC4431613

[46] Lima-Junior JC Jiang J Rodrigues-da-Silva RN Banic DM Tran TM Ribeiro RY B cell epitope mapping and characterization of naturally acquired antibodies to the *Plasmodium vivax* merozoite surface protein-3alpha (PvMSP-3alpha) in malaria exposed individuals from Brazilian Amazon. Vaccine (2011) 29(9):1801–11. 10.1016/j.vaccine.2010.12.099 21215342 PMC3065243

[47] Ladeia-Andrade S Ferreira MU Scopel KK Braga EM Bastos Mda S Wunderlich G Naturally acquired antibodies to merozoite surface protein (MSP)-1(19) and cumulative exposure to *Plasmodium falciparum* and *Plasmodium vivax* in remote populations of the Amazon Basin of Brazil. Mem Inst Oswaldo Cruz (2007) 102(8):943–51. 10.1590/S0074-02762007000800009 18209933

[48] Storti-Melo LM da Costa DR Souza-Neiras WC Cassiano GC Couto VS Povoa MM Influence of HLA-DRB-1 alleles on the production of antibody against CSP, MSP-1, AMA-1, and DBP in Brazilian individuals naturally infected with *Plasmodium vivax*. Acta Trop (2012) 121(2):152–5. 10.1016/j.actatropica.2011.10.009 22107686

[49] Braga EM Barros RM Reis TA Fontes CJ Morais CG Martins MS Association of the IgG response to *Plasmodium falciparum* merozoite protein (C-terminal 19 kD) with clinical immunity to malaria in the Brazilian Amazon region. Am J Trop Med Hyg (2002) 66(5):461–6. 12201577 10.4269/ajtmh.2002.66.461

[50] Baird JK. Age-dependent characteristics of protection v. susceptibility to *Plasmodium falciparum*. Ann Trop Med Parasitol (1998) 92(4):367–90. 10.1080/00034989859366 9683890

[51] Soe S Theisen M Roussilhon C Aye KS Druilhe P. Association between protection against clinical malaria and antibodies to merozoite surface antigens in an area of hyperendemicity in Myanmar: complementarity between responses to merozoite surface protein 3 and the 220-kilodalton glutamate-rich protein. Infect Immun (2004) 72(1):247–52. 10.1128/IAI.72.1.247-252.2004 14688102 PMC343946

[52] Oliveira-Ferreira J Pratt-Riccio LR Arruda M Santos F Daniel Ribeiro CT Goldberg AC HLA class II and antibody responses to circumsporozoite protein repeats of *P. vivax* (VK210, VK247 and *P. vivax*-like) in individuals naturally exposed to malaria. Acta Trop (2004) 92(1):63–9. 10.1016/j.actatropica.2004.02.011 15301976

[53] Yildiz Zeyrek F Palacpac N Yuksel F Yagi M Honjo K Fujita Y Serologic markers in relation to parasite exposure history help to estimate transmission dynamics of *Plasmodium vivax*. PLoS One (2011) 6(11):e28126. 10.1371/journal.pone.0028126 22140521 PMC3226671

[54] Modiano D Petrarca V Sirima BS Luoni G Nebie I Diallo DA Different response to *Plasmodium falciparum* in west African sympatric ethnic groups: possible implications for malaria control strategies. Parassitologia (1999) 41(1–3):193–7. 10697855

[55] Modiano D Chiucchiuini A Petrarca V Sirima BS Luoni G Roggero MA Interethnic differences in the humoral response to non-repetitive regions of the *Plasmodium falciparum* circumsporozoite protein. Am J Trop Med Hyg (1999) 61(4):663–7. 10548307 10.4269/ajtmh.1999.61.663

[56] Brisebarre A Kumulungui B Sawadogo S Afridi S Fumoux F Rihet P. Genome-wide significant linkage to IgG subclass responses against *Plasmodium falciparum* antigens on chromosomes 8p22-p21, 9q34 and 20q13. Genes Immun (2015) 16(3):187–92. 10.1038/gene.2014.66 25521226

[57] Afridi S Atkinson A Garnier S Fumoux F Rihet P Malaria resistance genes are associated with the levels of IgG subclasses directed against *Plasmodium falciparum* blood-stage antigens in Burkina Faso. Malar J (2012) 11:308 10.1186/1475-2875-11-308 22947458 PMC3552815

[58] Beck HP Felger I Barker M Bugawan T Genton B Alexander N Evidence of HLA class II association with antibody response against the malaria vaccine SPF66 in a naturally exposed population. Am J Trop Med Hyg (1995) 53(3):284–8. 7573714

[59] Banic DM Goldberg AC Pratt-Riccio LR De Oliveira-Ferreira J Santos F Gras-Masse H Human leukocyte antigen class II control of the immune response to p126-derived amino terminal peptide from *Plasmodium falciparum*. Am J Trop Med Hyg (2002) 66(5):509–15. 12201584 10.4269/ajtmh.2002.66.509

[60] Nardin EH Oliveira GA Calvo-Calle JM Castro ZR Nussenzweig RS Schmeckpeper B Synthetic malaria peptide vaccine elicits high levels of antibodies in vaccinees of defined HLA genotypes. J Infect Dis (2000) 182(5):1486–96. 10.1086/315871 11023472

[61] Murillo LA Rocha CL Mora AL Kalil J Goldenberg AK Patarroyo ME Molecular analysis of HLA DR4-beta 1 gene in malaria vaccinees. Typing and subtyping by PCR technique and oligonucleotides. Parasite Immunol (1991) 13(2):201–10. 10.1111/j.1365-3024.1991.tb00275.x 2052406

[62] Stephens HA Brown AE Chandanayingyong D Webster HK Sirikong M Longta P The presence of the HLA class II allele DPB1*0501 in ethnic Thais correlates with an enhanced vaccine-induced antibody response to a malaria sporozoite antigen. Eur J Immunol (1995) 25(11):3142–7. 10.1002/eji.1830251123 7489755

[63] Maitland K Williams TN Bennett S Newbold CI Peto TE Viji J The interaction between *Plasmodium falciparum* and *P. vivax* in children on Espiritu Santo island, Vanuatu. Trans R Soc Trop Med Hyg (1996) 90(6):614–20. 10.1016/S0035-9203(96)90406-X 9015495

[64] Luxemburger C Thwai KL White NJ Webster HK Kyle DE Maelankirri L The epidemiology of malaria in a Karen population on the western border of Thailand. Trans R Soc Trop Med Hyg (1996) 90(2):105–11. 10.1016/S0035-9203(96)90102-9 8761562

[65] Kaneko A Chaves LF Taleo G Kalkoa M Isozumi R Wickremasinghe R Characteristic age distribution of *Plasmodium vivax* infections after malaria elimination on Aneityum Island, Vanuatu. Infect Immun (2014) 82(1):243–52. 10.1128/IAI.00931-13 24166950 PMC3911855

[66] Bouharoun-Tayoun H Druilhe P. *Plasmodium falciparum* malaria: evidence for an isotype imbalance which may be responsible for delayed acquisition of protective immunity. Infect Immun (1992) 60(4):1473–81. 1548071 10.1128/iai.60.4.1473-1481.1992 PMC257020

[67] Chelimo K Ofulla AV Narum DL Kazura JW Lanar DE John CC. Antibodies to *Plasmodium falciparum* antigens vary by age and antigen in children in a malaria-holoendemic area of Kenya. Pediatr Infect Dis J (2005) 24(8):680–4. 10.1097/01.inf.0000172151.28851.fd 16094220

[68] John CC Moormann AM Pregibon DC Sumba PO McHugh MM Narum DL Correlation of high levels of antibodies to multiple pre-erythrocytic *Plasmodium falciparum* antigens and protection from infection. Am J Trop Med Hyg (2005) 73(1):222–8. 16014863

[69] Stanisic DI Richards JS McCallum FJ Michon P King CL Schoepflin S Immunoglobulin G subclass-specific responses against *Plasmodium falciparum* merozoite antigens are associated with control of parasitemia and protection from symptomatic illness. Infect Immun (2009) 77(3):1165–74. 10.1128/IAI.01129-08 19139189 PMC2643653

[70] Aribot G Rogier C Sarthou JL Trape JF Balde AT Druilhe P Pattern of immunoglobulin isotype response to *Plasmodium falciparum* blood-stage antigens in individuals living in a holoendemic area of Senegal (Dielmo, west Africa). Am J Trop Med Hyg (1996) 54(5):449–57. 8644897 10.4269/ajtmh.1996.54.449

[71] Metzger WG Okenu DM Cavanagh DR Robinson JV Bojang KA Weiss HA Serum IgG3 to the *Plasmodium falciparum* merozoite surface protein 2 is strongly associated with a reduced prospective risk of malaria. Parasite Immunol (2003) 25(6):307–12. 10.1046/j.1365-3024.2003.00636.x 14507328

[72] Nebie I Diarra A Ouedraogo A Soulama I Bougouma EC Tiono AB Humoral responses to *Plasmodium falciparum* blood-stage antigens and association with incidence of clinical malaria in children living in an area of seasonal malaria transmission in Burkina Faso, West Africa. Infect Immun (2008) 76(2):759–66. 10.1128/IAI.01147-07 18070896 PMC2223475

[73] Shi YP Sayed U Qari SH Roberts JM Udhayakumar V Oloo AJ Natural immune response to the C-terminal 19-kilodalton domain of *Plasmodium falciparum* merozoite surface protein 1. Infect Immun (1996) 64(7):2716–23. 8698500 10.1128/iai.64.7.2716-2723.1996 PMC174131

[74] Roussilhon C Oeuvray C Muller-Graf C Tall A Rogier C Trape JF Long-term clinical protection from falciparum malaria is strongly associated with IgG3 antibodies to merozoite surface protein 3. PLoS Med (2007) 4(11):e320. 10.1371/journal.pmed.0040320 18001147 PMC2071934

[75] John CC Tande AJ Moormann AM Sumba PO Lanar DE Min XM Antibodies to pre-erythrocytic *Plasmodium falciparum* antigens and risk of clinical malaria in Kenyan children. J Infect Dis (2008) 197(4):519–26. 10.1086/526787 18275273 PMC2607240

[76] Mishra S Nussenzweig RS Nussenzweig V. Antibodies to *Plasmodium* circumsporozoite protein (CSP) inhibit sporozoite’s cell traversal activity. J Immunol Methods (2012) 377(1–2):47–52. 10.1016/j.jim.2012.01.009 22306356 PMC3310221

[77] Stewart MJ Nawrot RJ Schulman S Vanderberg JP. *Plasmodium berghei* sporozoite invasion is blocked in vitro by sporozoite-immobilizing antibodies. Infect Immun (1986) 51(3):859–64. 3512436 10.1128/iai.51.3.859-864.1986 PMC260977

[78] Reineke U Sabat R. Antibody epitope mapping using SPOT peptide arrays. Methods Mol Biol (2009) 524:145–67. 10.1007/978-1-59745-450-6_11 19377943

[79] Lima-Junior JC Banic DM Tran TM Meyer VS De-Simone SG Santos F Promiscuous T-cell epitopes of *Plasmodium* merozoite surface protein 9 (PvMSP9) induces IFN-gamma and IL-4 responses in individuals naturally exposed to malaria in the Brazilian Amazon. Vaccine (2010) 28(18):3185–91. 10.1016/j.vaccine.2010.02.046 20189487 PMC2861348

[80] Lin HH Zhang GL Tongchusak S Reinherz EL Brusic V. Evaluation of MHC-II peptide binding prediction servers: applications for vaccine research. BMC Bioinformatics (2008) 9(Suppl 12):S22. 10.1186/1471-2105-9-S12-S22 19091022 PMC2638162

[81] Bueno LL Lobo FP Morais CG Mourao LC de Avila RA Soares IS Identification of a highly antigenic linear B cell epitope within *Plasmodium vivax* apical membrane antigen 1 (AMA-1). PLoS One (2011) 6(6):e21289 10.1371/journal.pone.0021289 21713006 PMC3119695

[82] Bergmann-Leitner ES Chaudhury S Steers NJ Sabato M Delvecchio V Wallqvist AS Computational and experimental validation of B and T-cell epitopes of the in vivo immune response to a novel malarial antigen. PLoS One (2013) 8(8):e71610. 10.1371/journal.pone.0071610 23977087 PMC3745447

[83] Lin M McRae H Dan H Tangorra E Laverdiere A Pasick J. High-resolution epitope mapping for monoclonal antibodies to the structural protein Erns of classical swine fever virus using peptide array and random peptide phage display approaches. J Gen Virol (2010) 91(Pt 12):2928–40. 10.1099/vir.0.023259-0 20810747

[84] Dyrberg T Oldstone MB Peptides as antigens. Importance of orientation. J Exp Med (1986) 164(4):1344–9. 10.1084/jem.164.4.1344 3760779 PMC2188429

[85] Jimah JR Salinas ND Sala-Rabanal M Jones NG Sibley LD Nichols CG Malaria parasite CelTOS targets the inner leaflet of cell membranes for pore-dependent disruption. Elife (2016) 5:e20621. 10.7554/eLife.20621 27906127 PMC5132341

