# Supplementary figures and images for: Plasmodium vivax Cell-Traversal Protein for Ookinetes and Sporozoites: Naturally Acquired Humoral Immune Response and B-Cell Epitope Mapping in Brazilian Amazon Inhabitants
Source: Front Immunol. 2017 Feb 7;8:77. doi: 10.3389/fimmu.2017.00077 (PMC5293784; doi:10.3389/fimmu.2017.00077)

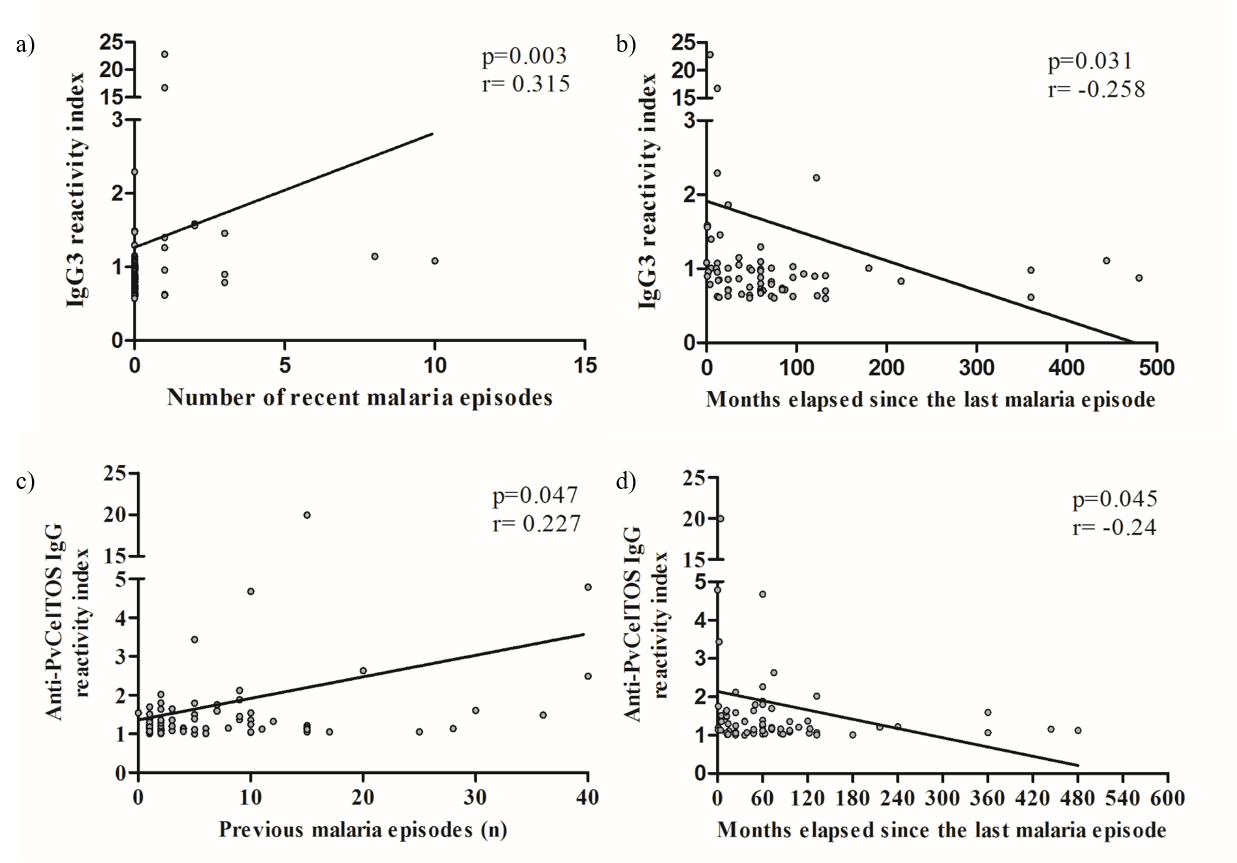

Supplement: Figure S1 — Associations of humoral response and exposition parameters in responders to PvCelTOS. (A) Spearman correlation between IgG3 reactivity index (RI) and number of recent malaria episodes; (B) Spearman correlation between IgG3 and months elapsed since the last malaria episode; (C) Spearman correlation between anti-PvCelTOS IgG reactivity index and number of previous malaria episodes; and (D) Spearman correlation between anti-PvCelTOS IgG RI and months elapsed since the last malaria episode. [file Image_1.TIF]
